# Factors involved in treatment durability and immunological recovery in a cohort of HIV-positive patients receiving atazanavir-based regimens

**DOI:** 10.7448/IAS.17.4.19830

**Published:** 2014-11-02

**Authors:** Andrea Giacomelli, Letizia Oreni, Marco Franzetti, Valentina Di Cristo, Barbara Vergani, Manuela Morosi, Elisa Colella, Massimo Galli, Stefano Rusconi

**Affiliations:** Infectious Diseases Unit, DIBIC Luigi Sacco, Milan, Italy

## Abstract

**Introduction:**

Since antiretroviral therapy must be taken lifelong, persistence and safety have become the goals to achieve. Protease inhibitors, in particular atazanavir (ATV) with or without ritonavir (r), represent a highly prescribed class in real life long-term treatment.

**Methods:**

We conducted a retrospective cohort study in HIV-1-positive patients who were followed at the Infectious Diseases Unit, DIBIC Luigi Sacco, University of Milan. Data regarding viral load, CD4 lymphocytes and the mean blood chemistry parameters were collected at baseline, first, third, sixth months from the beginning of therapy and then every six months. Factors related to persistence of therapy with ATV and time-dependent probability to reach a CD4 cells count >500 cells/µL were evaluated with Kaplan-Meier curve and Cox model.

**Results:**

A total of 1030 patients were evaluated: 183 received therapy with ATV/r as naïve, 653 switched to ATV/r as a second or following line and 194 switched to unboosted ATV from previous ATV-free regimens. A total of 138 patients shifted to unboosted ATV from a previous ATV/r regimen (17 from naïve ATV/r and 121 from experienced ATV/r). The median duration of therapy was 38 months (95% CI 29–73) in ATV/r naïve patients, 36 months (95% CI 23–53) in unboosted ATV group and 35 months (95% CI 31–43) in patients switched to ATV/r. We observed no significant difference in the persistence of the three regimens (p=0.149). Female (HR=1.317; 95% CI 1.073–1.616 p=0.008) and patients with CD4<200 cells/µL at baseline (HR=1.433 95% CI 1.086–1.892 p=0.011) were at increased risk of regimen interruption, whereas starting therapy with a backbone containing abacavir (HR=0.725; 95% CI 0.533–0.987 p=0.041) resulted protective. In multivariate analysis no significant difference between the three regimens was observed regarding reaching a count of CD4 cells >500 cells/µL. Factors associated to a poor CD4 gain were each extra Log of viral load at baseline (HR=0.915; 95% CI 0.852–0.982 p=0.014) and CD4<200 cells/µL at ATV start (HR=0.197; 95%CI 0.138–0.281 p<0.0001); conversely, females (HR=1.262; 95%CI 1.032–1.543 p=0.023) had a higher probability of CD4 recovery.

**Conclusions:**

Antiretroviral regimens containing atazanavir with or without ritonavir were durable and well tolerated, an elevated viral load and CD4 <200 cells/µL at baseline resulted related to regimen discontinuation and reduced CD4 recovery.

